# Knock-Down of Both *eIF4E1* and *eIF4E2* Genes Confers Broad-Spectrum Resistance against Potyviruses in Tomato

**DOI:** 10.1371/journal.pone.0029595

**Published:** 2011-12-29

**Authors:** Marianne Mazier, Fabrice Flamain, Maryse Nicolaï, Verane Sarnette, Carole Caranta

**Affiliations:** Unité de Génétique et Amélioration des Fruits et Légumes, INRA, UR1052, Montfavet, France; Ecole Normale Superieure, France

## Abstract

**Background:**

The eukaryotic translation initiation factor eIF4E plays a key role in plant-potyvirus interactions. eIF4E belongs to a small multigenic family and three genes, *eIF4E1*, *eIF4E2* and *eIF(iso)4E*, have been identified in tomato. It has been demonstrated that eIF4E-mediated natural recessive resistances against potyviruses result from non-synonymous mutations in an eIF4E protein, which impair its direct interaction with the potyviral protein VPg. In tomato, the role of eIF4E proteins in potyvirus resistance is still unclear because natural or induced mutations in *eIF4E1* confer only a narrow resistance spectrum against potyviruses. This contrasts with the broad spectrum resistance identified in the natural diversity of tomato. These results suggest that more than one eIF4E protein form is involved in the observed broad spectrum resistance.

**Methodology/Principal Findings:**

To gain insight into the respective contribution of each eIF4E protein in tomato-potyvirus interactions, two tomato lines silenced for both *eIF4E1* and *eIF4E2* (RNAi-4E) and two lines silenced for *eIF(iso)4E* (RNAi-iso4E) were obtained and characterized. RNAi-4E lines are slightly impaired in their growth and fertility, whereas no obvious growth defects were observed in RNAi-iso4E lines. The F1 hybrid between RNAi-4E and RNAi-iso4E lines presented a pronounced semi-dwarf phenotype. Interestingly, the RNAi-4E lines silenced for both *eIF4E1* and *eIF4E2* showed broad spectrum resistance to potyviruses while the RNAi-iso4E lines were fully susceptible to potyviruses. Yeast two-hybrid interaction assays between the three eIF4E proteins and a set of viral VPgs identified two types of VPgs: those that interacted only with eIF4E1 and those that interacted with either eIF4E1 or with eIF4E2.

**Conclusion/Significance:**

These experiments provide evidence for the involvement of both eIF4E1 and eIF4E2 in broad spectrum resistance of tomato against potyviruses and suggest a role for eIF4E2 in tomato-potyvirus interactions.

## Introduction

Plant viruses are obligatory intracellular parasites that infect many economically important crops and cause severe economic losses. Among the techniques available to counter viral infections, one of the most effective and sustainable approach is the deployment of genetic resistance targeted directly against viruses. Over the past several years, there have been dramatic advances in our understanding of the molecular nature and mechanisms underlying natural resistances. Dominant and recessive resistance genes have been characterized at the molecular level and new principles of innate viral immunity associated with gene silencing are currently emerging paving the way for new strategies to better exploit and promote the use of genetic resistances [1]–[3].

A significant breakthrough in natural resistance gene mechanisms was achieved by demonstrating the key role of translation initiation factors eIF4E, and to a lesser extent eIF4G, in plant resistance to RNA viruses [4]. eIF4E binds to the 5′ cap structure of mRNA and also to eIF4G to form the eIF4F complex. Additional translation initiation factors and the ribosomal 40S subunit are then recruited to initiate mRNA translation [5]. Higher plants are unique in that they encode two distinct isoforms of eIF4F that have both overlapping and isoform-specific roles: eIF4F, which contains eIF4E and eIF4G, and eIF(iso)4F, which contains eIF(iso)4E and eIF(iso)4G [6]–[8]. Although these two complexes are considered equivalent for the *in vitro* translation of some mRNAs, they differ in their *in vivo* expression patterns and demonstrate some specificity for different capped cellular mRNAs [7], [8]. In dicotyledons, several genes code for eIF4E and eIF4G proteins. In *Arabidopsis*, for example, three genes code for proteins of the eIF4E subfamily and one codes for eIF(iso)4E. In tomato, 2 genes code for eIF4E proteins, and one codes for eIF(iso)4E [9]. Although eIF4E has been implicated in resistance to several viral genera [10]–[11], the majority of eIF4E-mediated resistances function against viruses belonging to the genus *Potyvirus*. This genus is one of the largest among plant viruses and causes considerable economic damages to many crop species. eIF4E-mediated recessive resistances against potyviruses result from a small number of amino acid changes in the eIF4E protein [4], [12]. The exact mechanism by which these mutations confer resistance is still unclear, but several results suggest the resistance is due to an altered binding with the potyviral protein VPg [13]. Therefore, a physical interaction between wild type eIF4E (hereafter referred to as the susceptibility allele) and viral VPg is required for viral infection, and amino acid changes in the eIF4E protein encoded by the resistance allele impair binding with VPg and prevent infection [14].

Besides natural resistances which are not always present in the genetic diversity of crop species, biotechnological approaches offer other means to limit viral diseases. Virus resistance obtained by transgenic techniques was one of the earliest commercialized biotech traits [15]. The majority of virus-resistant transgenic plants were obtained using the pathogen-derived resistance strategy mediated either by proteins or nucleic acids through RNA silencing (also known as RNA interference or RNAi) [16], [17]. An alternative strategy to engineer virus resistant plants is to target susceptibility genes (*i.e.*, genes encoding host factors required for the viral infection cycle), as the loss of the susceptibility functions of such genes should lead to resistance. For example, resistant transgenic tobacco plants have been obtained using RNAi directed against two host genes previously identified to support tobamovirus multiplication [18].

In addition to transgenic techniques, TILLING (Targeting Induced Local Lesions IN Genomes, [19]) technology is another straightforward and cost-effective way to obtain resistance to viruses through loss-of-function mutations. TILLING was successfully exploited to engineer potyvirus resistant tomato plants by targeting *eIF4E* genes [9]. An *eif4e1* null mutant (hereafter referred to as the *eif4e1* mutant) was demonstrated to be immune to a strain of *Potato virus Y* (PVY) and to *Pepper mottle virus* (PepMoV) and susceptible to other potyviruses. In comparison with previous results demonstrating broad spectrum resistance to potyviruses in the wild tomato relative *Solanum habrochaïtes* PI247087 involving eIF4E1 [20], it is striking that the *eif4e1* mutant shows a narrow resistance spectrum. These results suggest that some potyviruses may use more than one eIF4E protein to infect their hosts.

To gain insight into the respective contributions of eIF4E proteins into tomato-potyvirus interactions, a RNAi strategy was developed using constructs designed to silence either *eIF4E1* and *eIF4E2* or *eIF(iso)4E*. In this analysis, we show that the simultaneous RNAi-induced silencing of *eIF4E1* and *eIF4E2* confers broad spectrum resistance to potyviruses and identifies eIF4E2 as an additional plant factor involved in the outcome of tomato-potyvirus interactions.

## Results

### Generation of transgenic lines and specificity of the RNAi constructs toward *eIF4E* genes

**Figure 1:**
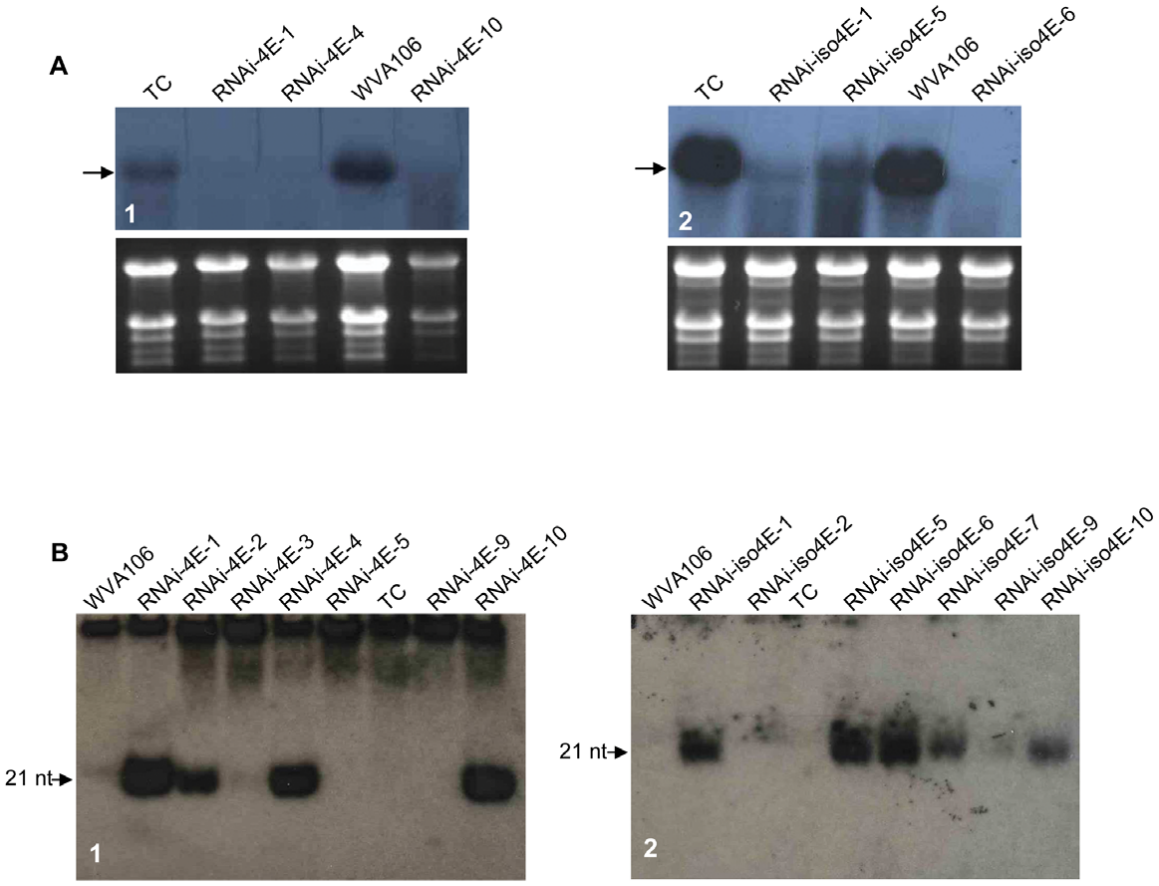
Northern blot analysis of primary transformants using transgene specific-probes. (**A**) Northern blot analysis of total RNA. (A1) Hybridization with the *eIF4E1* full length cDNA. (A2) Hybridization with the *eIF(iso)4E* full length cDNA. The expected size for the transcript is indicated by an arrow. RNA load was assessed by gel electrophoresis and ethidium bromide staining (bottom panels). (**B**) Northern blot analysis of low-molecular-weight RNA. (B1) Hybridization with a 200-bp *eIF4E1* specific probe that does not overlap with the RNAi-4E construct. (B2) Hybridization with a 200-bp *eIF(iso)4E* specific probe that does not overlap with the RNAi-iso4E construct. The signal corresponding to the 21-nt siRNA is indicated with an arrow. TC corresponds to transgenic control (WVA106 transformed with an empty vector).

To investigate the respective contributions of each eIF4E protein in tomato-potyvirus interactions, a RNAi strategy was developed to silence either *eIF4E1* and *eIF4E2*, which share 82% identity in their cDNA sequences, or *eIF(iso)4E*. Successful silencing in the putative primary transformants was assessed by northern blot analysis of total RNA (Figure 1A) and low-molecular-weight RNA (Figure 1B). The following independent homozygous single T-DNA insertion plants were selected for further experiments: RNAi-4E-1 and RNAi-4E-10, silenced for *eIF4E* expression; and RNAi-iso4E-1 and RNAi-iso4E-6, silenced for *eIF(iso)4E* expression.

**Figure 2:**
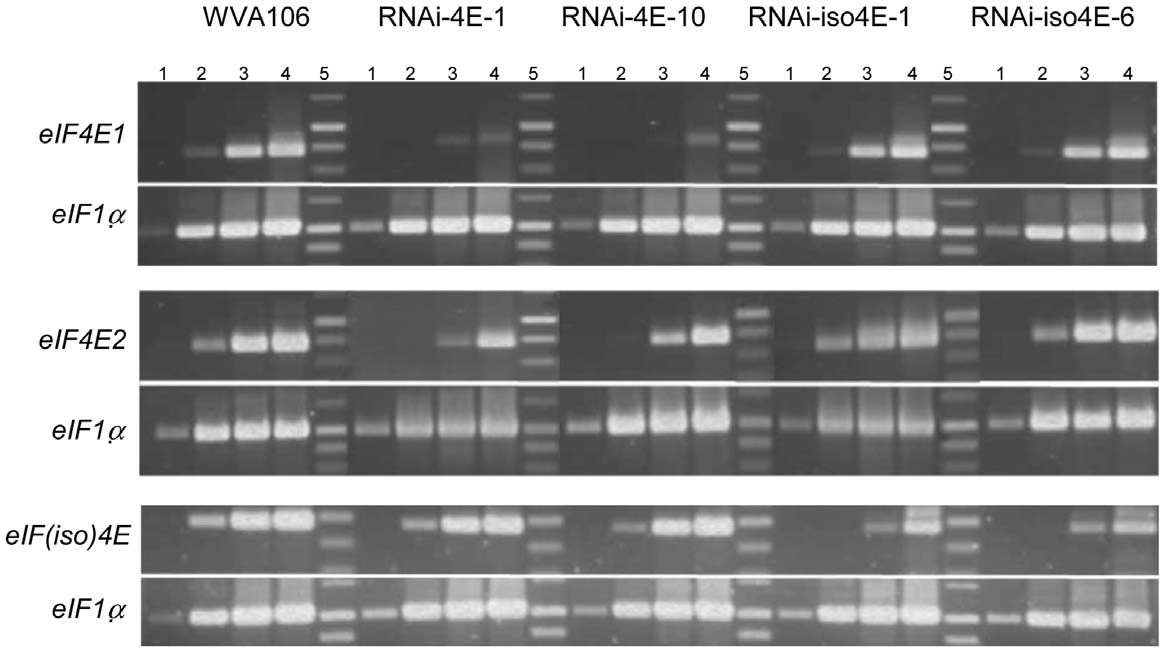
Comparison of the relative accumulation of *eIF4E1*, *eIF4E2* and *eIF(iso)4E* transcripts in transgenic lines by semi-quantitative RT-PCR. For each eIF4E transcript, the elongation factor elF1α was used as control. The reactions were sampled after 20 (lane 1), 25 (lane 2), 30 (lane 3) and 35 (lane 4) cycles for each genotype. Lane 5: molecular weight.

To determine the silencing spectrum for the *eIF4E* genes, semi-quantitative RT-PCR experiments were performed using gene specific primers (Figure 2). A decrease in *eIF4E1* and, to a lesser extent, in *eIF4E2* transcript accumulation was detected for RNAi-4E-1 and RNAi-4E-10 lines in comparison with WVA106. No significant decrease in *eIF4E1* and *eIF4E2* accumulation was detected in the RNAi-iso4E-1 and RNAi-iso4E-6 lines. Conversely, a decrease in *eIF(iso)4E* accumulation was detected for the RNAi-iso4E-1 and RNAi-iso4E-6 lines but not for RNAi-4E-1 and RNAi-4E-10 lines. Together these results indicate that the RNAi-4E construct induces silencing of both *eIF4E1* and *eIF4E2* but does not silence *eIF(iso)4E*, whereas the RNAi-iso4E construct induces the specific silencing of *eIF(iso)4E*.

### Silencing of *eIF4E* genes impairs growth and fertility

**Figure 3:**
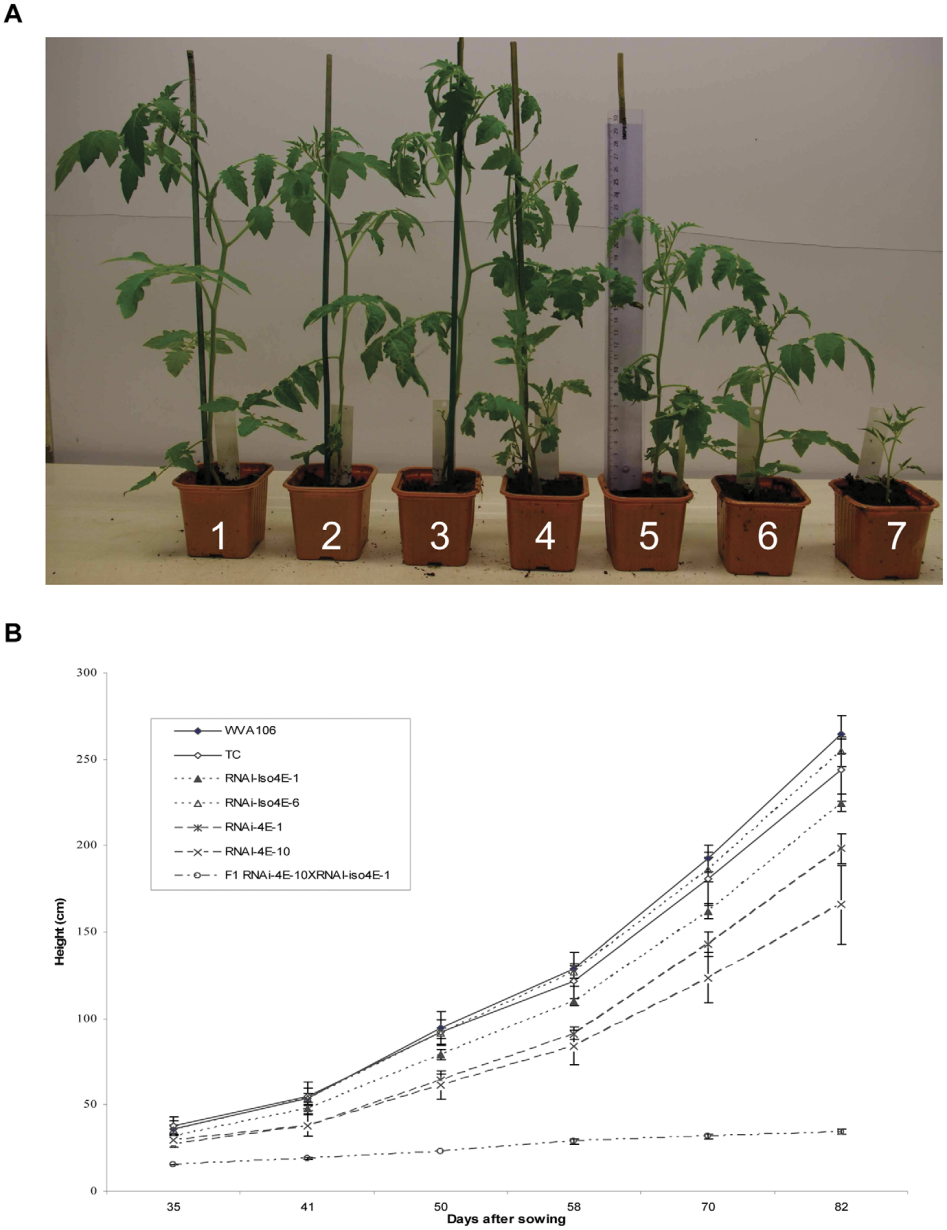
Transgenic lines silenced for several *eIF4E* genes are impaired in their growth. (**A**) Representative plants at 30 days after sowing for (1) WVA106, (2) transgenic control (TC), (3) RNAi-iso4E-1, (4) RNAi-iso4E-6, (5) RNAi-4E-1, (6) RNAi-4E-10 and (7) F_1_(RNAi-4E-10×RNAi-iso4E-1). Seeds were germinated in soil and grown in growth chambers with a day length of 16 h, at 24°C during day and 18°C during night. (**B**) Growth rate of WVA106, transgenic lines and F_1_(RNAi-4E-10×RNAi-iso4E-1) from 35 to 82 days after sowing. Plants were grown under standard greenhouse conditions. Each data point represents the mean stem height of 3 plants per line. Error bars represent the standard mean deviation.

Although the phenotypes of RNAi-iso4E-1 and RNAi-iso4E-6 lines showed no obvious vegetative defects in comparison with WVA106 and transgenic controls (hereafter named TC), the RNAi-4E-1 and RNAi-4E-10 plants consistently presented a semi-dwarf phenotype (Figure 3A). To further characterize the impact of eIF4E silencing on plant development and fertility, RNAi-4E-10 was crossed with RNAi-iso4E-1, and the resulting F1 progeny were grown along with parental and control lines on soil under standard greenhouse conditions. Effectiveness of RNAi in the F1 progeny was confirmed using semi-quantitative RT-PCR (Figure S2). The growth rates of the F1 plants were significantly reduced compared to the WVA106 and TC plants (Figure 3B). Significant differences in terminal plant height were also observed. The RNAi-4E-1 and RNAi-4E-10 plants sized 75% and 62% of WVA106 respectively, whereas the F1(RNAi-4E-10×RNAi-iso4E-1) showed an even more pronounced phenotype (13% of WVA106 in height). No delay in flowering time was recorded between the eIF4E silenced plants and the control plants and all of the genotypes produced fruits.

**Figure 4:**
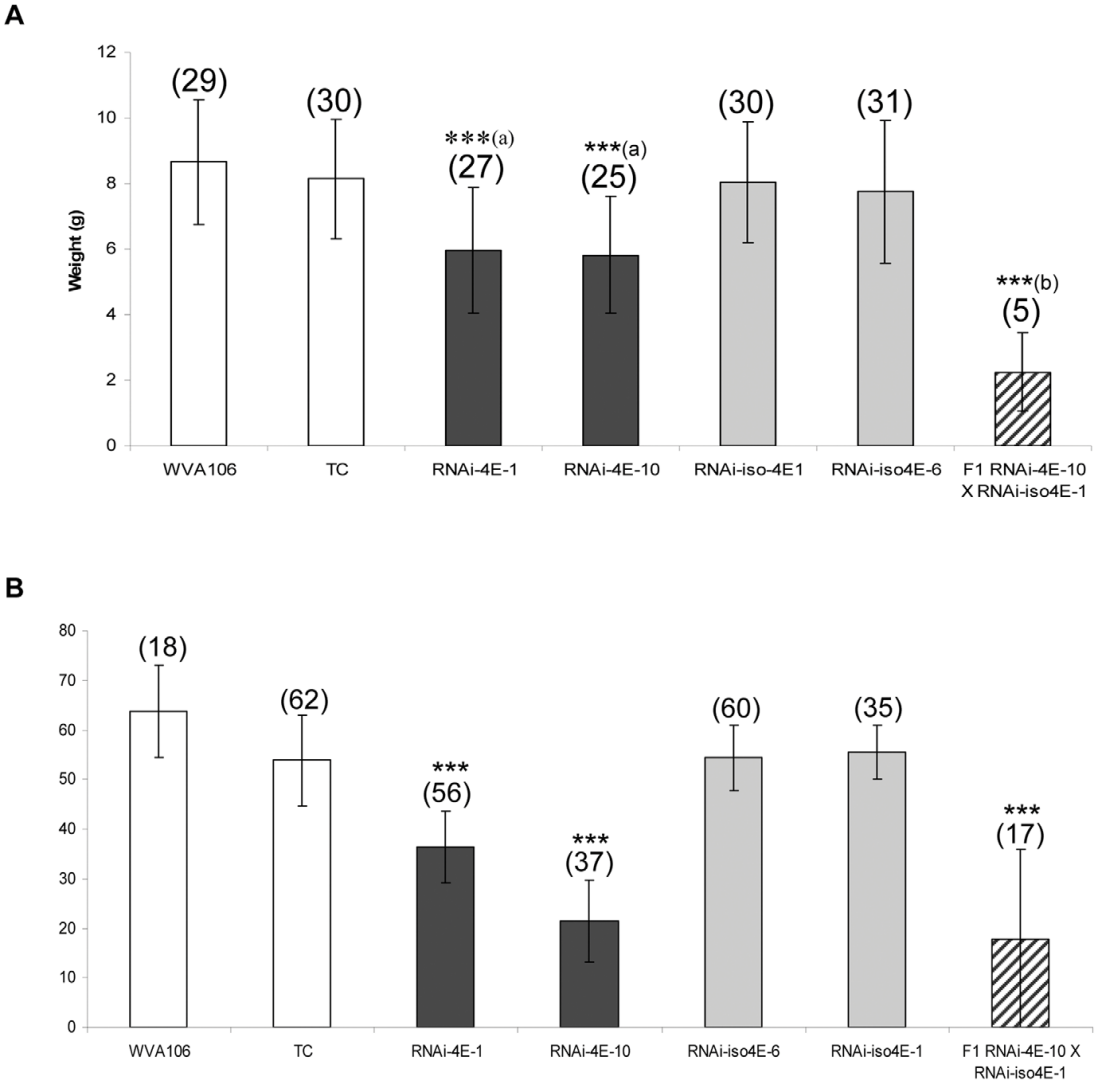
Transgenic lines silenced for several *eIF4E* genes are affected in their fertility. (**A**) Average fruit weight and number of fruits per plants. Fruits from three plants per lines were harvested and individually weighed. The numbers in brackets represent the number of fruits harvested per plants. Kruskal-Wallis statistical tests were performed to identify significant differences between genotypes. ***a and ***b indicate significant differences (P<0.05). (**B**) Average number of seeds per fruit. Numbers in brackets correspond to the number of fruits analyzed for their seeds content. *** indicate significant differences (P<0.05).

Fruits from three plants per genotype were individually weighted, and the number of seeds per fruits was counted. While no significant differences were detected between RNAi-iso4E lines and the controls, the fruits harvested from RNAi-4E lines were significantly smaller and lighter, and a more pronounced phenotype was observed in the F1(RNAi-4E-10×RNAi-iso4E-1) hybrid (Figure 4A). The number of seeds per fruits was reduced in the RNAi-4E lines and the F1(RNAi-4E-10×RNAi-iso4E-1) hybrid (Figure 4B). On the whole, the RNAi-4E lines presented a semi-dwarf phenotype and their fertility was affected. The F1(RNAi-4E×RNAi-iso4E) hybrid presented an even more pronounced phenotype, consisting of dwarf plants with thread-like leaves that produced a few small fruits, suggesting a cumulative effect of the silencing of *eIF4E* and *eIF(iso)4E* genes.

### Transgenic lines silenced for both *eIF4E1* and *eIF4E2* genes are resistant to several potyviruses

**Figure 5:**
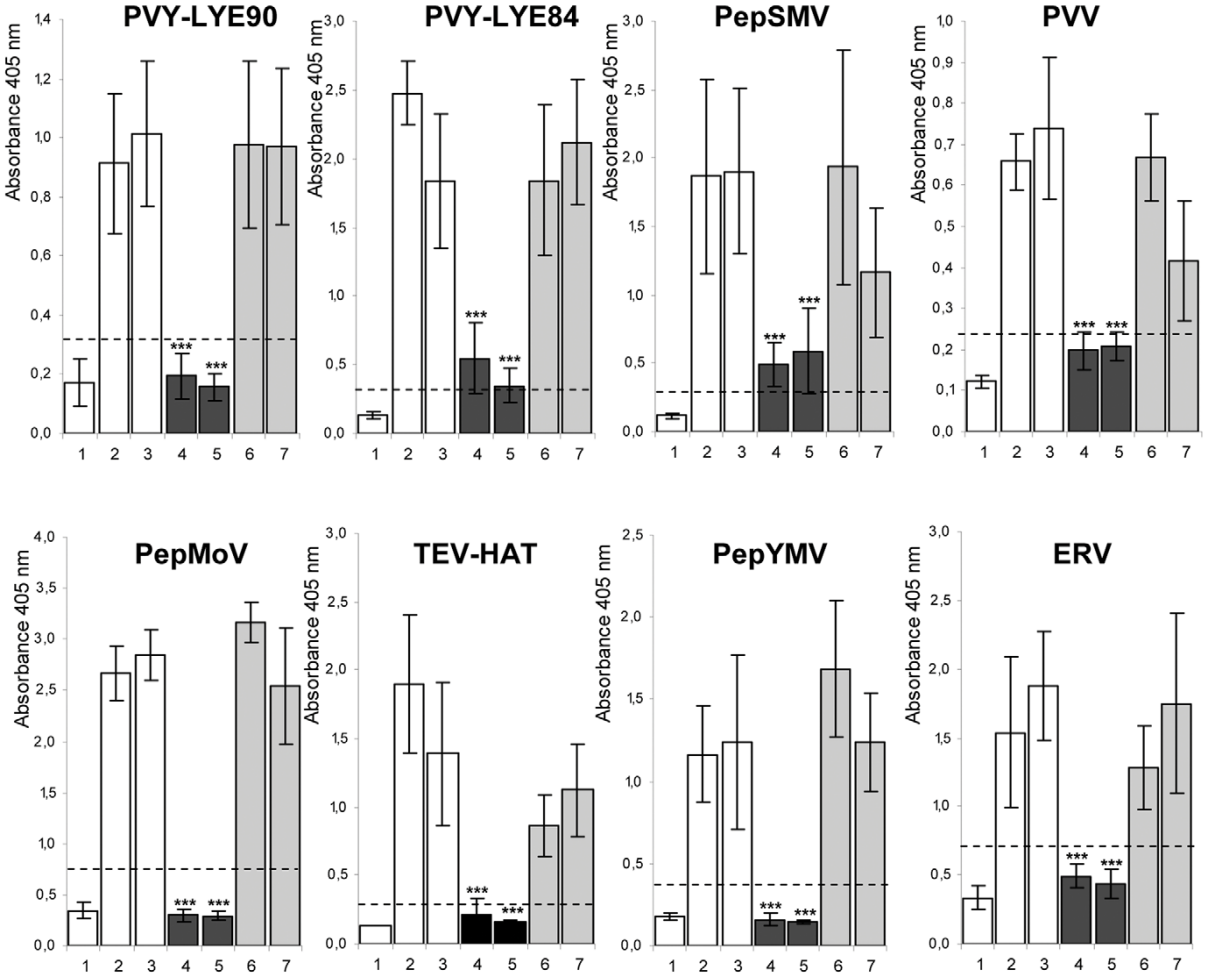
Effect of the knockdown of *eIF4E1/eIF4E2* or *eIF(iso)4E* expression on potyvirus accumulation. Viral coat protein accumulation of TEV-HAT, PVY-LYE84, PVY-LYE90, PepSMV, PVV, PepMoV-Tex, ERV and PepYMV assessed by DAS-ELISA in systemic leaves at 15 to 18 days post inoculation (dpi) using a potyvirus specific antibody (“Potyvirus Group”, Agdia, France). 1: Healthy control (non inoculated WVA106 plants); 2: Susceptible control (inoculated WVA106 plants); 3: Transgenic control (WVA106 transformed with an empty vector); 4: RNAi-4E-1; 5: RNAi-4E-10; 6: RNAi-iso4E-1; 7: RNAi-iso4E-6. A dotted bar represents 2 times the absorbance value obtained with healthy controls. The error bars indicate standard error. Kruskal-Wallis statistical tests were performed to identify significant differences between genotypes. *** indicate significant differences (P<0.05).

To examine whether the silencing of *eIF4E* or *eIF(iso)4E* genes has an impact on the outcome of viral infection, homozygous T_2_ from RNAi-4E-1, RNAi-4E-10, RNAi-iso4E-1 and RNAi-iso4E-6 lines were challenged with strains of the following 7 potyviruses: PVY, TEV, PepMoV, *Ecuadorian rocotto virus* (ERV), *Pepper severe mosaic virus* (PepSMV), *Pepper yellow mosaic virus* (PepYMV), and *Potato virus V* (PVV). The following members of other viral genera were also used to chanllenge the tomato lines: *Tomato spotted wilt virus* (TSWV), *Alfalfa mosaic virus* (AMV), *Cucumber mosaic virus* (CMV) and *Tobacco mosaic virus* (TMV). The reactions of the transgenic lines, WVA106 and TC, against the viral strains were determined by mechanical inoculation of 18 plants per genotypes during three independent assays. Because the majority of the potyviruses do not induce obvious symptoms on tomato, resistance/susceptibility was assessed using DAS-ELISA. For all tested potyvirus strains, virus accumulation was obvious at 15 to 18 days post-inoculation (dpi) in the WVA106 and TC lines, but no viral accumulation or levels significantly lower than those observed in the controls was observed for the RNAi-4E-1 and RNAi-4E-10 lines (Figure 5). Partial resistance rather than complete immunity was observed after inoculation with the PVY-LYE84 and PepSMV strains. This result is most likely due to the fact that the silencing of *eIF4E1* and *eIF4E2* genes in the RNAi-4E lines is not complete. During all of the experiments, the RNAi-iso4E-1 and RNAi-iso4E-6 plants were fully susceptible to all potyviruses and statistically indistinguishable from inoculated WVA106 and TC plants. These results demonstrate that the silencing of both the *eIF4E1* and *eIF4E2* genes in a susceptible tomato genotype confers resistance to a broad range of potyviruses. Moreover, the observation that all RNAi lines were fully susceptible to other viral genera, including TSWV, AMV, CMV and TMV (Table S1), demonstrates the specificity of eIF4E-mediated resistance toward potyviruses.

### The VPg of some potyviral strains interacts with both eIF4E1 and eIF4E2

**Figure 6:**
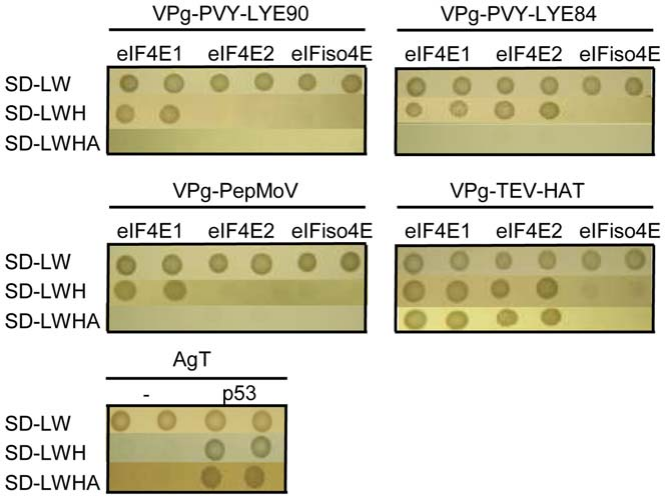
Interaction assays between the three eIF4E proteins from tomato and a selected set of VPg from potyviruses using the yeast two-hybrid system. Yeast expressing both “bait” and “prey” recombinant proteins were obtained by first transforming yeast cells with the individual plasmid construction followed by separate conjugation between yeasts expressing eIF4E proteins and those expressing VPg proteins. Conjugations were then cultured on control plates (SD-LW) and on two selective media lacking leucine, tryptophan and histidine (SD-LWH) or leucine, tryptophan, histidine and adenine (SD-LWHA). Each plasmid combination was spotted in duplicate. Positive and negative controls from the Matchmaker GAL4 two-hybrid system 3 were used (bottom panel).

The broad spectrum resistance to potyviruses observed in RNAi lines silenced for both *eIF4E1* and *eIF4E2*, when compared with the narrow resistance spectrum of the *eif4e1* mutant [9], suggests the involvement of both proteins in tomato-potyvirus interactions. To test this hypothesis and because previous results have shown that physical interaction between eIF4E and VPg is necessary for viral infection [13], [14], we performed yeast two-hybrid interaction assays between the three eIF4E proteins from susceptible tomato lines and a selected set of VPg proteins. Three independent protein-protein interaction experiments were carried out, each conducted in duplicate. No interaction could be detected between eIF(iso)4E and any of the VPg proteins whereas eIF4E1 interacted with all the VPg proteins that were tested. The interaction between eIF4E1 and the TEV-HAT VPg is likely to be stronger because the yeasts transformed with both partners grew on the more stringent media lacking both adenine and histidine. In contrast, eIF4E2 displayed a specific interaction pattern depending on the VPg protein: eIF4E2 interacted with VPg from PVY-LYE84 and TEV-HAT but failed to interact with either the VPg from PVY-LYE90 or the VPg from PepMoV (Figure 6). Consequently, two types of viral strains were identified with respect to VPg/eIF4E interaction: PVY-LYE90 and PepMoV which interact only with eIF4E1, and PVY-LYE84 and TEV-HAT which interact with both eIF4E1 and eIF4E2.

## Discussion

This study exploiting RNAi to down-regulate the expression of eIF4E translation initiation factors provides evidence for the involvement of both eIF4E1 and eIF4E2 in broad spectrum resistance of tomato against potyviruses. To gain insight into the respective contributions of *eIF4E1*, *eIF4E2* and *eIF(iso)4E* in tomato-potyvirus interactions, two hairpin RNAi constructs for either *eIF4E1/eIF4E2* or *eIF(iso)4E* were used to stably transform *S. lycopersicon* cv. WVA106. We show that simultaneous RNAi-induced silencing of both *eIF4E1* and *eIF4E2* confers resistance to a wide range of potyviruses, including PVY and TEV, which is the most harmful to tomato cultivation. In comparison with previous work showing that the *eif4e1* mutant obtained by TILLING is resistant to a narrow range of potyvirus strains [9], this work demonstrates that the targeting of both *eIF4E1* and *eIF4E2* grants broad spectrum resistance against potyviruses. The involvement of eIF4E in the viral infectious cycle appears to be restricted to potyviruses, as infection by TSWV, AMV, CMV and TMV is not impaired in transgenic lines silenced for *eIF4E* or *eIF(iso)4E*.

Previous results have shown that the physical interaction between eIF4E and VPg is necessary for viral infection [13], [14] and that some potyvirus strains are able to use several eIF4E proteins for infection [21]–[23]. Consequently, we investigated whether the broad spectrum resistance identified in the RNAi-4E lines that were silenced for *eIF4E1* and *eIF4E2* could be explained by the capacities of potyviral strains to use several eIF4E proteins. Protein-protein interaction assays between eIF4Es and VPgs support this hypothesis. In addition, these results explain the differences in the resistance spectrum observed between RNAi-4E lines and the *eif4e1* mutant. The RNAi-4E lines are resistant to all potyviral strains, whereas the *eif4e1* mutant is resistant to PVY-LYE90 and to PepMoV but susceptible to PVY-LYE84 and TEV-HAT [9]. We show that the VPgs of PVY-LYE90 and PepMoV only interact with eIF4E1, while the VPgs of PVY-LYE84 and TEV-HAT interact with both eIF4E1 and eIF4E2. These results demonstrate a perfect match between the patterns of eIF4E/VPg interactions and the resistance/susceptibility phenotype observed in RNAi-4E lines and the *eif4e1* mutant. The fact that none of the VPgs interacted with eIF(iso)4E is in agreement with the observed susceptibility of the RNAi lines silenced for *eIF(iso)4E* and rules out the involvement of eIF(iso)4E in tomato-potyvirus interactions.

Previously, significant breakthroughs concerning the role of eIF4E in plant resistance to potyviruses were obtained through the characterization of the natural polymorphism of *eIF4E* genes. In solanaceous crops and many other species, it was demonstrated that natural resistance to potyviruses results from amino acid changes in the protein encoded by the recessive resistance allele [12]. Functional analyses conducted in pepper (*Capsicum annuum*) demonstrated that these amino acid changes mediate resistance by disrupting the direct interaction with the viral VPg [13], [14]. However, it remains uncertain whether the outcome of the eIF4E-VPg interaction is the sole determinant of potyviral infectivity for many plant-potyvirus pairs [12]. In tomato, for example, natural amino acid changes identified in the eIF4E1 protein encoded by the *pot1* resistance allele did not impair physical interaction with potyviral VPgs ([20], Gallois et al., unpublished data). These results, together with the data obtained in the present study, suggest that a more complex scheme might operate in tomato, and they strongly support a role for eIF4E2 in the outcome of tomato-potyvirus interaction. The observation that the *eif4e2* TILLING mutant (with a stop codon mutation in exon 1) is susceptible to potyviruses [9] indicates that the knock-out of *eIF4E2* is not sufficient to interfere with potyviral infection and therefore suggests a role for eIF4E2 that superimposes to the role of eIF4E1.

The mechanism by which eIF4E2 is involved in tomato-potyvirus interactions remains to be elucidated, but the absence of mutations in the coding sequence of *eIF4E2* between resistant and susceptible tomato genotypes (Caranta et al., unpublished data) suggests a mechanism different from the well-documented mechanism involving amino acid changes. One possible mechanism might be that the respective amounts of eIF4E1 and eIF4E2 proteins constitute an additional parameter in the outcome of the interaction. It has been previously shown that eIF4E protein level accumulation is tightly regulated across the gene family. Post-translational regulations are thought to compensate for the lack of expression of one of the genes of the eIF4E-encoding family. In *Arabidopsis thaliana*, for example, eIF4E over- accumulates in an *At-eifiso4e* genetic background [24]. This accumulation would be part of the mechanism that allows different *eIF4E* genes to compensate for one another to maintain the essential cellular function of eIF4E, which is host protein synthesis [6], [24]. Therefore, we could hypothesize that in the *eif4e1* mutant, eIF4E2 would over-accumulate and substitute for eIF4E1 towards PVY-LYE84 and TEV-HAT but not towards PVY-LYE90 or PepMoV. In the RNAi-4E lines, both the eIF4E1 and eIF4E2 protein levels are decreased, allowing a broad spectrum of resistance. The compensatory effect between eIF4E1 and eIF4E2 is also supported by the fact that the RNAi lines silenced for both genes are slightly impaired in their growth and fertility, whereas no obvious growth defects were observed neither in the *eif4e1* and *eif4e2* TILLING mutants [9] nor in RNAi lines silenced for *eIF(iso)4E* (this study).

### Conclusion

In conclusion, these experiments provide evidence for the involvement of both eIF4E1 and eIF4E2 in the broad spectrum resistance of tomato against potyviruses and suggest a role for eIF4E2 in tomato-potyvirus interactions. The exact mechanism by which eIF4E2 is involved in tomato-potyvirus interactions remains to be elucidated but our results suggest that the amount of eIF4E1 and eIF4E2 proteins could constitute an additional parameter determining the outcome of the interaction.

## Materials and Methods

### Transgene constructions and production of transgenic tomatoes

To obtain the *eIF4E1* and *eIF(iso)4E* specific cDNA fragments, reverse transcription (RT) was performed using 2 µg of total RNA extracted using TRI Reagent (Euromedex, France). A 310-bp fragment corresponding to the 5′ half (positions 79–389) of the *eIF4E1* cDNA sequence (GenBank accession AY723733) and a 200-bp fragment spanning the middle part (positions 296–496) of the *eIF(iso)4E* cDNA sequence (GenBank accession BT014561) were amplified by PCR using the gene-specific primers 5′GGGGACAAGTTTGTACAAAAAAGCAGGCTGATGGAGGAGGAGGAGAGGT and 5′GGGGACCACTTTGTACAAGAAAGCTGGGTTCCCACTGTGGCTCAATTTT for *eIF4E1* and 5′GGGGACAAGTTTGTACAAAAAAGCAGGCTTGCGGACTTTCATTTGTTCA and 5′GGGGACCACTTTGTACAAGAAAGCTGGGTCCTACGCACACTAGCAACCA for *eIF(iso)4E*. These primers contained both attB1 and attB2 recombination sites (underlined). The purified DNA fragments were cloned as inverted repeats under the control of the 35S promoter using the Gateway cloning system as described by Karimi et al. [25] to obtain the following hairpin RNAi constructs: RNAi-4E for the specific silencing of *eIF4E1* and *eIF4E2* and RNAi-iso4E for the specific silencing of *eIF(iso)4E*. The constructs were checked by sequencing and used to transform *Solanum lycopersicum* cv West Virginia 106 (WVA106, susceptible to viruses) by the *Agrobacterium tumefaciens* strain C58pGV2260. WVA106 was also transformed with an empty vector (named TC for transgenic control). Tomato genetic transformation was performed according to Hamza and Chupeau [26] using MSO medium [27] complemented with 0.9 mg/l thiamine, 0.2 mg/l 2–4D, 0.1 mg/l kinetin and 0.2 mM Acetosyringone, and a regeneration medium (MSO medium completed with 2 mg/l zeatin) supplemented with antibiotics (100 mg/l kanamycin and 225 mg/l timentin) until regeneration of buds. The regenerated buds were transplanted individually onto elongation medium (MSO medium in which MS salts were reduced to ½) containing 100 mg/l kanamycin and 225 mg/l timentin until rooting. Growth chamber conditions were 22°C during the day, 18°C during the night with a day length of 16 h. T_1_ transgenic progenies obtained by self-pollination of primary transformants confirmed to be silenced for *eIF4E* or *eIF(iso)4E* gene expression were assessed for kanamycin resistance to select T_1_ plants with a single active kanamycin locus. Two independent lines selected from each RNAi construct, namely RNAi-4E-1, RNAi-4E-10, RNAi-iso4E-1 and RNAi-iso4E-6, each showed a 3∶1 ratio consistent with the segregation of a single kanamycin resistance locus. Homozygous kanamycin resistant T_2_ lines were used for phenotypic analysis.

### Molecular characterization of transgenic plants

Total genomic DNA was isolated from leaves using a modified CTAB method, and Southern blot analysis was performed as described in Dubois et al. [28], [29]. Single transgene insertion was confirmed by Southern blot analysis, and the expression of the kanamycin transgene was verified by northern blot analysis (Figure S1). Total RNAs were extracted from 200–300 mg of young tomato leaves using TRI Reagent (Euromedex, France). Northern blot analysis of total RNA was performed as described in Dubois et al. [29]. Hybridizations were performed using the *eIF4E1* or *eIF(iso)4E* full length cDNAs. RNA gel blot analysis of low-molecular-weight RNA was resolved using 15 µg of total RNA, as described in Dunoyer et al. [30]. Hybridizations were performed using 200-bp probes specific for *eIF4E1* or *eIF(iso)4E* that did not overlap with the RNAi-4E or RNAi-iso4E constructs, respectively. Radiolabelled specific probes were obtained by random priming reactions with 40 ng of purified DNA fragments in the presence of α^32^P dCTP (Amersham, France).

For semi-quantitative RT-PCR, first strand cDNAs were synthesized from 2.5 µg of total RNA in 20-µl reactions with 100 µM oligodT_21_ and avian myeoloblastosis virus reverse transcriptase (AMV reverse transcriptase, Promega, France) using standard procedures. Specific amplification of the 700-bp *eIF4E1* cDNA was performed using 5′CTGAAATGGAGAGAACGATGT and 5′CACTGCATCAAGAACTATACGG primers. Specific amplification of the 700-bp *eIF4E2* cDNA was performed using 5′GGGACGAAAACACCAAAAATG and 5′CCCTGTTGTAACGATAGAACTA primers, and specific amplification of the 670-bp *eIF(iso)4E* cDNA was performed using 5′GCACCGTAGAGGCGACGGAG and 5′GCAGCTCAGATGGGCATTGG primers. Specific amplifications of eIF4E cDNAs and elongation factor *eIF1α* control were carried out in separate tubes treated in parallel during the same PCR experiment under the following conditions: 94°C for 3 minutes, then 20, 25, 30 and 35 PCR cycles at 94°C for 15 s, 52°C for 30 s and 72°C for 60 s. RT-PCR products were separated on a 1% agarose gel.

### Virus strains and infection assays

Potyvirus infection assays were performed using PVY-LYE84 [31], PVY-LYE90 [32], TEV-HAT [33], ERV [34], PepSMV [35], PepYMV [36], PepMoV-Texas [37] and PVV [38]. Strains were maintained in *Nicotiana benthamiana*. The viruses from other genera, TSWV-LYE51, AMV-LYE80 and TMV-SM-1 were maintained in *Solanum lycopersicum* and CMV-I17F was maintained in *Cucumis melo*.

Four-week-old T_2_ plants per genotypes were mechanically inoculated as described in Ruffel et al. [20]. Inoculated plants were maintained in a growth chamber with a day length of 16 h, at 24°C during the day and 18°C during the night. Viral accumulation in systemic leaves was tested by DAS-ELISA at 15 to 18 days post-inoculation (dpi) for all viruses (except for TSWV and TMV for which obvious symptoms were observed) using antisera specific for potyviruses (“potyvirus group”, Agdia France) or for AMV or CMV (provided by the plant Pathology Unit, UR407 INRA-Avignon France). The Kruskal-Wallis non-parametric test was used to identify significant differences between genotypes [39].

### Yeast two-hybrid analysis

*eIF4E* and *VPg* coding sequences were amplified by PCR using high-fidelity Platinium Taq polymerase from oligo(DT)-primed reverse transcription products. Gene-specific primers were designed to introduce restriction enzyme sites. The VPg cistron was amplified from the NIa PCR product using a reverse primer incorporating a stop codon at the end of the coding sequence. PCR products were cloned into a pGEMT-easy vector (Promega) and sequenced. Coding sequences were then subcloned in-frame with the GAL4 activation domain or the GAL4 binding domain into the pGADT7 or pGBKT7 vectors (Clontech). All pGADT7- and pGBKT7-derived vectors were sequenced using primer T7 to check orientation and in-frame insertion. *eIF4E* coding sequences corresponding to *eIF4E1*, *eIF4E2* and *eIF(iso)4E* were amplified from *Solanum lycopersicum* cv. M82.

The yeast two-hybrid assay was performed as described by Charron et al. [14] using the Matchmaker GAL4 two-hybrid system 3 (Clontech), except that pGADT7- and pGBKT7-derived vectors were transformed into PJ69 4a and PJ69 4α yeast strains. The growth of yeast colonies containing both prey and bait vectors was used as a control on synthetic medium lacking leucine and tryptophan (SD-LT). Interaction between prey and bait was tested at both low (medium lacking leucine, tryptophan and histidine; SD-LTH) and high (medium lacking leucine, tryptophan, histidine and adenine; SD-LTHA) stringencies, and each combination was shown in duplicate. The expression in yeast of the three tomato eIF4E proteins was confirmed by western blot analysis (data not shown).

## Supporting Information

Figure S1 **Analysis of kanamycin resistant T1 trangenic tomato genotypes.** (**A**) Southern blot analysis. DNAs were digested with *Xba*I and transferred to nylon membranes for hybridization with the *nptII* probe labelled with ^32^P-dCTP. (**B**) Northern blot analysis with the *nptII* probe. The expected size for the transcript is indicated with an arrow. Ethidium bromide-stained gel was used (bottom panel) as loading control. TC corresponds to transgenic control (WVA106 transformed with an empty vector).
(TIF)

Figure S2 **Accumulation of** ***eIF4E1*** **and** ***eIF(iso)4E*** **transcripts in the F1(RNAi-4E-10×RNAi-iso4E-1) by semi-quantitative RT-PCR.** Reactions were sampled after 20 (lane 1), 25 (lane 2), 30 (lane 3) and 35 (lane 4) cycles for each genotype. Lane 5: molecular weight. Elongation factor elF1α was used as control.
(TIF)

Table S1 **Behavior of RNAi-4E and RNAi-iso4E lines towards other viral genera.** Number of susceptible plants/number of inoculated plants. Viral infection was assessed by visual evaluation of symptoms for TSWV-LYE51 and TMV-SM1 and by DAS-ELISA at 18 dpi for AMV-LYE80 and CMV-I17F.
(DOC)
