# Supplementary figures and images for: Knock-Down of Both eIF4E1 and eIF4E2 Genes Confers Broad-Spectrum Resistance against Potyviruses in Tomato
Source: PLoS One. 2011 Dec 29;6(12):e29595. doi: 10.1371/journal.pone.0029595 (PMC3248445; doi:10.1371/journal.pone.0029595)

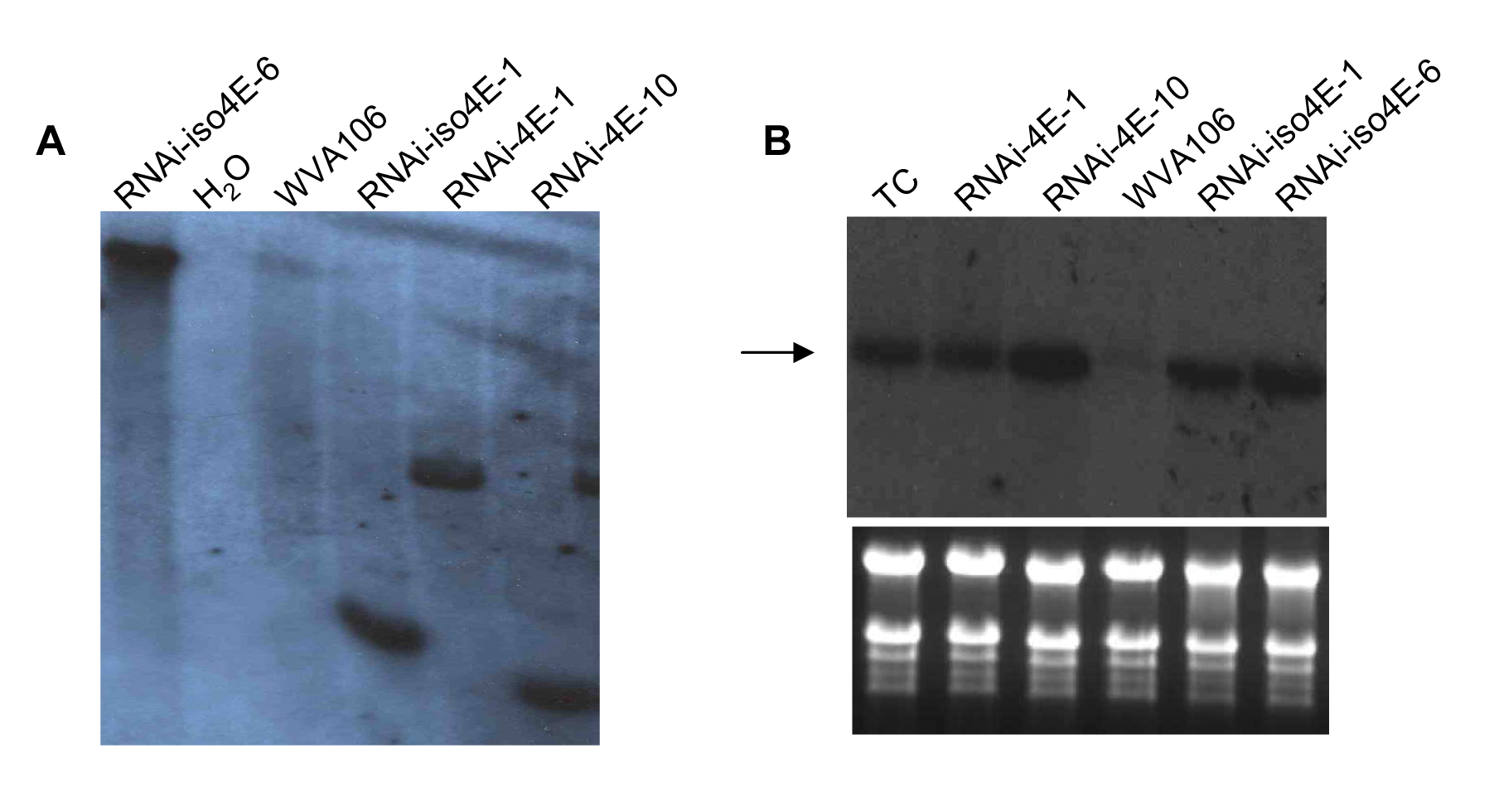

Supplement: Figure S1 — Analysis of kanamycin resistant T1 trangenic tomato genotypes. (A) Southern blot analysis. DNAs were digested with XbaI and transferred to nylon membranes for hybridization with the nptII probe labelled with 32P-dCTP. (B) Northern blot analysis with the nptII probe. The expected size for the transcript is indicated with an arrow. Ethidium bromide-stained gel was used (bottom panel) as loading control. TC corresponds to transgenic control (WVA106 transformed with an empty vector). (TIF) [file pone.0029595.s001.tif]

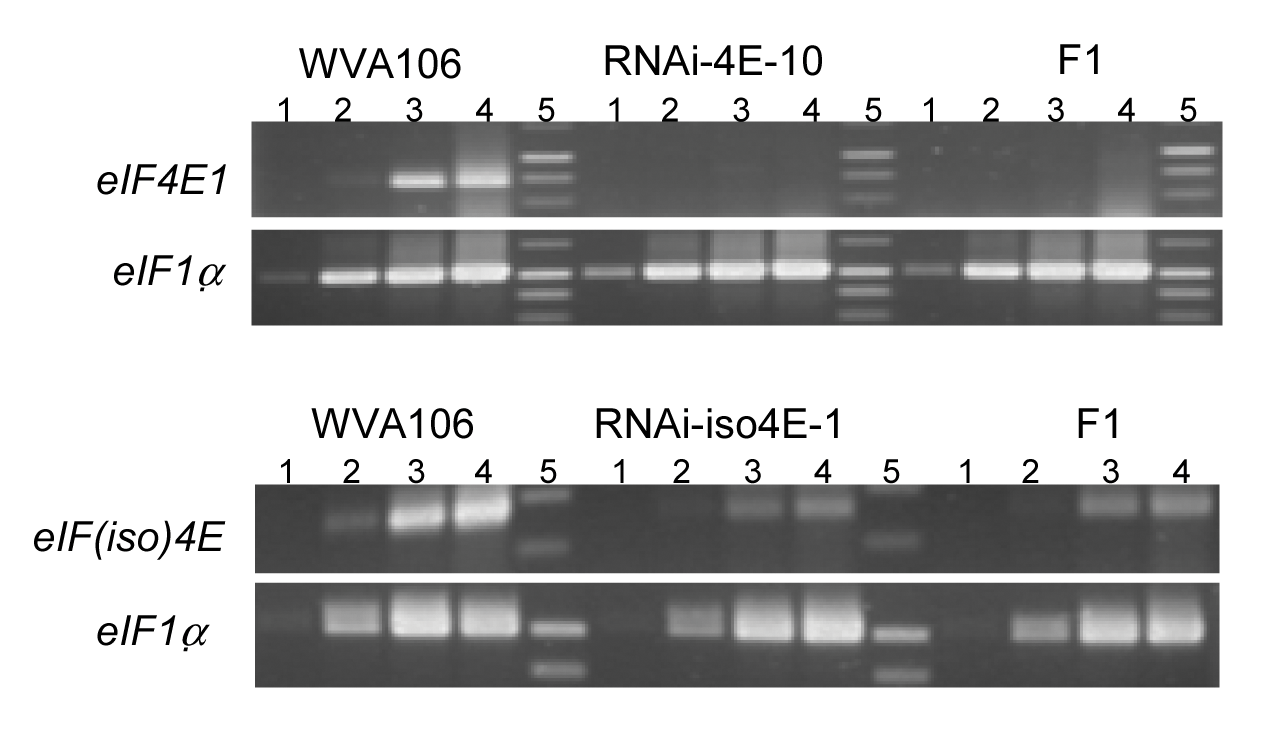

Supplement: Figure S2 — Accumulation of eIF4E1 and eIF(iso)4E transcripts in the F1(RNAi-4E-10×RNAi-iso4E-1) by semi-quantitative RT-PCR. Reactions were sampled after 20 (lane 1), 25 (lane 2), 30 (lane 3) and 35 (lane 4) cycles for each genotype. Lane 5: molecular weight. Elongation factor elF1α was used as control. (TIF) [file pone.0029595.s002.tif]
