# Supplementary material for: Knock-Down of Both eIF4E1 and eIF4E2 Genes Confers Broad-Spectrum Resistance against Potyviruses in Tomato
Source: PLoS One. 2011 Dec 29;6(12):e29595. doi: 10.1371/journal.pone.0029595 (PMC3248445; doi:10.1371/journal.pone.0029595)
Supplement: Table S1 — Behavior of RNAi-4E and RNAi-iso4E lines towards other viral genera. Number of susceptible plants/number of inoculated plants. Viral infection was assessed by visual evaluation of symptoms for TSWV-LYE51 and TMV-SM1 and by DAS-ELISA at 18 dpi for AMV-LYE80 and CMV-I17F. (DOC) [file pone.0029595.s003.doc]

Table S3

|  | TSWV-LYE51 | AMV-LYE80 | CMV-I17F | TMV-SM1 |
| --- | --- | --- | --- | --- |
| Healthy control | 0/5 | 0/5 | 0/5 | 0/5 |
| WVA106 | 5/5 | 5/5 | 5/5 | 5/5 |
| TC | 5/5 | 5/5 | 5/5 | 5/5 |
| RNAi-4E-1 | 5/5 | 5/5 | 5/5 | 5/5 |
| RNAi-4E-10 | 5/5 | 5/5 | 5/5 | 5/5 |
| RNAi-iso4E-1 | 5/5 | 5/5 | 5/5 | 5/5 |
| RNAi-iso4E-6 | 5/5 | 5/5 | 5/5 | 5/5 |
